# Human touch? Acoustical analysis of ancient music reconstructs tuning and intonation, elucidating aspects of human behavior

**DOI:** 10.1126/sciadv.adv3101

**Published:** 2025-05-07

**Authors:** Dan C. Baciu

**Affiliations:** Professorship for Data Driven Design, Münster School of Architecture, Münster University of Applied Sciences, Münster, NRW, Germany.

## Abstract

Did you ever travel to Greece and wonder what ancient Greek or Roman music sounded like? A mathematical analysis of all compositions that have survived from antiquity now allows us to reconstruct the exact tuning and intonation. According to this analysis, ancient musicians preferred pure intonation. However, they had a keen sense of its combinatorial limitations on instruments with strings of fixed length, such as lyres, and they recognized the necessity of slight deviations from pure intonation during vocal performance to allow for more tonal complexity. This spirit is paralleled in ancient atomic philosophy, which posited that atoms sometimes swerved to allow for more combinatorial complexity and unexpected effects.

## Teaser

Mathematical analysis reconstructs tuning of ancient Greek and Roman music and reveals parallels with atomist philosophy.

## INTRODUCTION

**Fig. 1.**
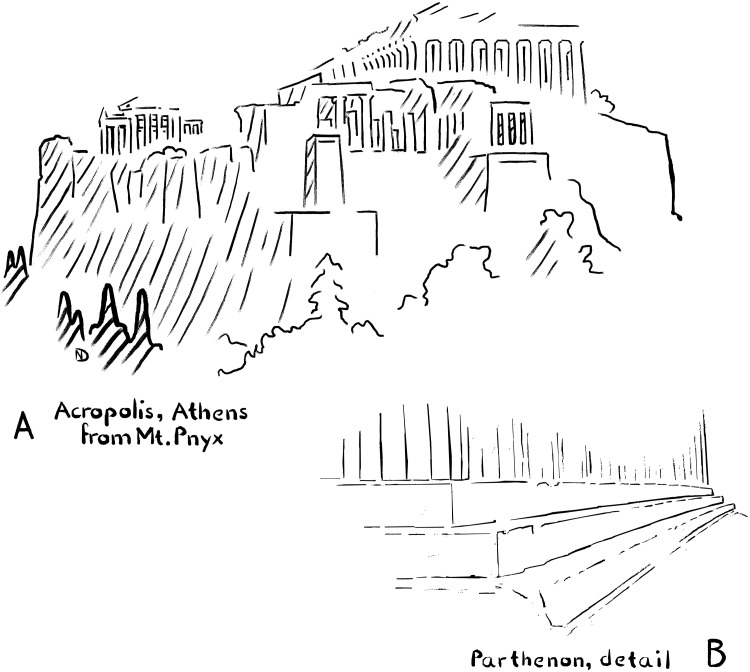
Linearity and slight curves in ancient Greek temples. Architect’s sketches. (**A**) From a distance, the Parthenon appears to be a rectilinear design. (**B**) Yet, close-up perspectives reveal that most lines, including those of the platform, are slightly bent, gently swaying away from linearity.

Ancient Greek and Roman art often comes across as natural yet highly sophisticated. For example, ancient Greek temples featured highly refined designs in which straight-looking lines are actually gently curved to convey a sense of life. Take the most famous temple on the Athenian Acropolis—the Parthenon. In principle, it is simply a rectangular building executed with the same type of columns all around. Yet, the design is highly elaborate: The platform on which the columns are erected is slightly bent, as if duplicating the curvature of the horizons (Fig. 1); the columns are tilted inward at different angles, and the column shafts are tapered toward the top while swelling slightly toward their centers (*1*).

Did the finesse of these gentle curves also exist in ancient Greek and Roman music? The answer provided here begins by bringing the cultural context into focus. In Classical Antiquity, two instruments were particularly prominent: the lyre and the auloi. The lyre is a type of instrument with ~7 fixed strings, each producing one distinct tone. The auloi are double pipes that can be used to play many different tones and gently modulate them, allowing for portamentos, melismas, and similar acoustic swerves.

Lyres and auloi not only function in different ways, but they have also been associated with different kinds of music. For centuries, ancient Greek mythology has connected the lyre with Apollon, a farsighted god, whereas the auloi were more closely associated with Dionysos, a free-spirited god of frenzied or chaotic behavior, sometimes referred to as Liber. Apollon was also the holder of the bow, which allowed him to act at a distance. Dionysos was the god of wine who influenced things on the go. This distinct symbolism still reverberated in the 19th century when the philosopher Friedrich Nietzsche developed a whole theory of Apollonian and Dionysian modes of artistic expression, permeating all ancient as well as modern cultures. The “Apollonian” was lofty, ordered, and perfected. The “Dionysian” was captivating, challenging, and romantic—just like the music Nietzsche himself liked to compose.

Ancient musicians knew not only of the distinction between lyres and auloi and between different gods, but they also made use of two entirely different musical notation styles. These two styles are mostly referred to as “instrumental” and “vocal” notation. For consistency, these terms will be used throughout the article.

**Fig. 2.**
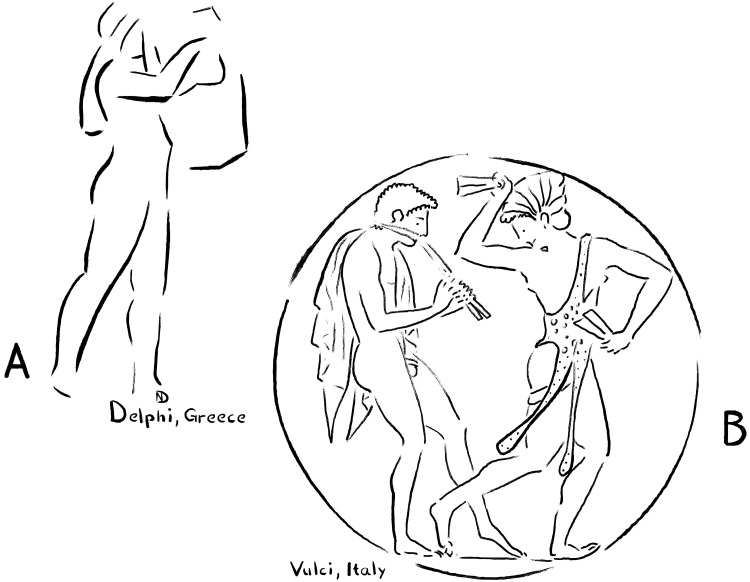
Comparison of lyre and auloi. (**A**) Performer with a so-called “kithara,” as chiseled on the Athenian Treasury in Delphi. Whenever two strings were played one after the other on this type of instrument, their sound briefly overlapped. With pure intonation, the resulting sound was harmonious and constant. In this illustration, the pure acoustics of the compositions are reflected in the performer’s upright posture. On the same corner of the Athenian Treasury, one can also find the hymn of Limenios, which is perhaps the most remarkable instrumental composition that survives from antiquity. It was inscribed there in 128 BCE and starts with the words, “Come to the farsighted…” (**B**) Compositions for wind instruments or unaccompanied voice involved more tonal complexity and playful nonlinearity, requiring the adoption of melismas or portamentos. The same playfulness is also reflected in the postures of the performers shown in this illustration, which depicts an auloi player in a vivid encounter with a dancer (*5*). Nonlinearity returns in Greek art and philosophy in the slight curves of temple architecture and in the swerving behavior that philosophers and poets attributed to atoms.

The main discovery reported here begins with the observation that there is a perfect match on one hand between lyres, Apollonian foresight, and music that has survived in instrumental notation and on the other hand between auloi, Dionysian free-flow, and music written in vocal notation. Here is what can be observed:

Analysis of music written in instrumental notation immediately reveals that this music was very likely composed for lyres. With only a few exceptions, instrumental pieces feature exactly seven distinct tones, which is also the number of strings that lyres are typically represented with. In addition, instrumental music makes excellent use of the resonance characteristics of lyres. By default, two lyre strings plucked in a sequence produce two tones that briefly resound together and interfere. As one tone still fades out, the other already begins to resound. If the wavelengths of these tones harmonize, the resulting tonal transition is experienced as pure and lofty. The present mathematical analysis reveals that ancient composers favored such clean transitions. Indeed, all instrumental pieces that have been passed down to us exhibit impeccable harmonic integrity. This achievement must have been a key feature of music composed for lyres. The tuning of lyre strings is fixed; it cannot be adjusted during the performance. Therefore, avoiding any kind of tuning compromise required careful composition and attention to harmonic constraints. The result is an exquisite kind of music with melodic lines that proceed from tone to tone almost as if following an oracle: Surprises may occur, yet they are always harmonically preordained, and they are most beautiful if performed on a flawlessly tuned instrument (Fig. 2A). These Apollonian qualities clearly distinguish instrumental compositions from those written in vocal notation.

Whereas instrumental compositions make for a perfect match with lyres and Apollonian foresight, compositions written in vocal notation make for a better match with auloi and Dionysian free-flow. Performing music on a pair of auloi allows for liberties unheard of in lyre performances. The present mathematical analysis demonstrates that music in vocal notation made use of these liberties, necessitating an instrument such as the auloi. The following example clearly illustrates the problem. Consider the subtle melody “do-so-re-fa-do.” It seems paradoxical, but if one uses pure intonation for this melody, one returns to the initial point slightly out of tune. The slight deviation is known in the literature as the “syntonic comma”—a small pitch discrepancy that can sometimes arise as a by-product of pure intonation. In music for lyres, no single sequence of tones is encountered that leads into a syntonic comma. However, syntonic commas are common in music written in vocal notation. As a result, the performers of these latter pieces had to modulate the tones somehow, in ways not necessarily predetermined, to make up for the deviations. Thus, this style of music comes through as less mathematically planned and more free-spirited, requiring the performers to adjust the intonation on the go. Tonal modulations such as melismas or portamentos could have been the response of performers when playing these complex compositions that go beyond the combinatorial limitations that were never transgressed in music for lyres (Fig. 2B).

The slight adjustments in pitch that the performers of vocal music had to adopt exhibit an interesting property. They slightly bend the melodic lines of the music. Why this is so will be explained in detail in the Results section of this article. For now, it is enough to note that these gentle curves and swerves could be seen as a musical analog to the soft curvatures found in temple architecture.

Perhaps the curved melodic lines also tell an additional, greater story. As it has been observed, ancient composers had a keen sense of the acoustical properties of sound. They were able to compose stunningly harmonic pieces that can be played on instruments with fixed strings, allowing for flawless intonation throughout. At the same time, composers opted to force the performer to slightly swerve with his or her voice to facilitate higher combinatorial complexity in pieces written in vocal notation. These vocal swerves, although small, may have sparked something quite remarkable. In the broader context of Antiquity, similar swerving behavior that breaks combinatorial limitations was not only required from singers but also a behavior attributed to atoms. Ancient Greek and Roman philosophers correctly inferred that our stunningly complex world is composed of limited types of invisibly small particles or “atoms.” Additionally, they foresaw that the atoms had to occasionally swerve, gently going off-path, to allow for more substantial complexity. This behavior was also used to explain the origins of free will in living beings.

Atomism is a philosophy that experienced remarkable success. Initially, it was inspired by the act of writing. Specifically, it was inspired by the idea that letters could be recombined into words and sentences, facilitating the creation of stunningly complex texts. Perhaps atomism was also inspired by the idea that musical tones could be combined and recombined into countless melodies. In addition, these melodies sometimes forced the performer to go off-pitch, slightly swerving with the voice to achieve a higher degree of musical complexity. Perhaps the necessity of this free-spirited swerving inspired philosopher-poets to propose that something just like it also existed in the motion of atoms.

## RESULTS

### How can music be described with numbers?

Already in antiquity, authors such as the Pythagoreans and Ptolemy believed that music had a mathematical beauty, and they translated music into mathematics. The basic principles of their translation are still valid today, having provided some of the first theoretical foundations for the field of acoustics.

The present article briefly returns to the Pythagoreans and Ptolemy. It uses the basic acoustical concepts that they initially developed to evaluate the musical compositions that have survived from antiquity. This approach is particularly worthwhile. It facilitates observations that make it possible to reconstruct how exactly these ancient musical compositions were tuned.

How, then, did the ancients translate music into mathematics? To explain their approach, two musical terms are relevant: tones and intervals. A tone is a single sound produced by a musical instrument or the human voice, characterized by its fundamental pitch or wavelength. Most music consists of tones. Nevertheless, a single tone is rarely considered to be true music. Music mostly comes to life through melodies, which consist of chains of multiple, different tones. In these chains, each transition between two tones is referred to as an interval. Thus, an interval can be defined as a sequence of two consecutive tones. Most musical styles gain their richness from the diversity of intervals that they use.

The question that the ancients already asked was whether there was a way to mathematically describe intervals. Take an “octave” as an example. To make an octave resound, one has to play two tones, with the second tone being an octave higher than the first. Yet, how large exactly is an octave? Is there a reproducible way to describe its exact size?

To answer this question, the ancients found an elegant solution—the monochord. In essence, a monochord is an instrument with a single string. To play any imaginable interval, one divides this string into two parts, the relationship of which can be described with a fraction. Let us return to the previous example of the octave: One can divide the string of the monochord into two parts, the first twice as long as the second. Then, plucking the first and second parts of the string one after the other, one obtains the octave. Thus, 1/2 stands for a pure octave. Technically, multiplying the length of the first part of the monochord string by 1/2, one receives the length of the second part.

In ancient Greek terms, an octave was called a “diapason” or “all-embracing” interval. In modern music, the name “octave” comes from the Latin term for eight. An octave is the size of eight consecutive tones on a modern piano. Other intervals are the major and minor thirds, the fourths and fifths, and the major and minor sixths. All of these intervals can be represented with fractions: 2/3 stands for a pure fifth, 3/4 for a pure fourth, and 4/5 and 5/6 for pure major and minor thirds, respectively, whereas 3/5 and 5/8 stand for pure sixths.

This type of mathematical translation of intervals was already discovered in antiquity. Since then, the acoustical principles on which it relies have stood the test of time. The representation of intervals with fractions has not only spearheaded the development of acoustics but has remained valid for millennia. Today, sound is understood as a vibration with characteristic frequencies and wavelengths. Here again, intervals can be described with fractions. An octave is composed of two tones, and the relationship between their fundamental wavelengths can be mathematically described by the fraction 1/2. The same type of description remains valid for all other intervals mentioned above.

This mathematical description of intervals is not only accurate in historical and modern terms, but it also allows for the formulation of more complex mathematical statements. In particular, fractions can be broken down into smaller fractions. For example, 1/2 = 2/3 × 3/4. This mathematical statement can be translated back into music. As mentioned above, 1/2 stands for an octave, whereas 2/3 and 3/4 stand for a pure fifth and fourth, respectively. Translated to music, the mathematical statement 1/2 = 2/3 × 3/4 means “an octave is as much as a fifth and a fourth together.”

A fifth is a bright and powerful interval, sometimes referred to as the “dominant.” It can be broken down even further into two smaller intervals: the major and minor thirds. The equation is 2/3 = 4/5 × 5/6. Translated to music, the equation states “a fifth is as much as a major and minor third together.”

The thirds are sweet-sounding intervals. Modern major and minor scales are distinguished by their distinct use of major and minor thirds.

As just demonstrated, mathematical notation can be used to describe the act of breaking down intervals into smaller intervals. We have broken down an octave of 1/2 into a fifth of 2/3 and a fourth of 3/4, writing this as 1/2 = 2/3 × 3/4. In turn, we broke down a fifth of 2/3 into a major third of 4/5 and a minor third of 5/6, writing this as 2/3 = 4/5 × 5/6. Additionally, octaves can also be broken down into thirds and sixths, which come in both major and minor flavors. Mathematically, one can state 1/2 = 4/5 × 5/8 and 1/2 = 3/5 × 5/6.

Once the intervals are translated into mathematics, all multiplications and divisions are allowed, and these operations have a clear physical meaning that can be translated back into music. For example, we have broken down a fifth into major and minor thirds. Mathematically, this was written 2/3 = 4/5 × 5/6. Obviously, we can switch this equation around, writing 4/5 × 5/6 = 2/3. The meaning is similar yet slightly different. Here, rather than breaking down a fifth into thirds, we combine thirds to make a fifth. The same can also be verbally stated as “going up a major and minor third, one reaches a fifth higher.”

Other operations are also possible. For instance, if one goes up a major third and down a minor third, one reaches only roughly a quarter tone higher. Mathematically, 4/5 × 6/5 = 24/25. Here, the fraction 6/5 indicates that one is going down the minor third rather than up; hence, the fraction is inverted. The interval 24/25 is very small. Human ears can hardly distinguish it from the mathematically adjacent 23/24 or 25/26. In our analysis, we shall therefore focus on intervals that are at least as large and distinguishable as a minor third (5/6).

As has just been established, there are large and small intervals. In addition, intervals can be played in different ways, slightly larger or smaller. In technical terms, this is referred to as “intonation.” There are two types of intonation: pure and impure. If an interval can be represented as a fraction composed of small integers, its intonation can be referred to as “pure intonation.” Pure intonation comes naturally to many performers, and it has an important acoustical advantage. It arranges intervals in harmonic ways. As a result, pure intonation creates a constant harmonic sound.

**Fig. 3.**
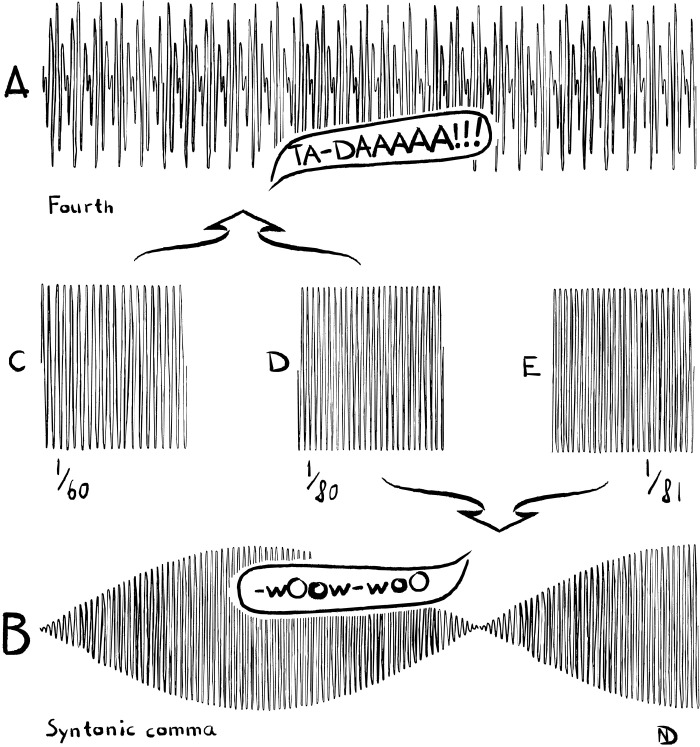
Interference patterns in pure and impure intervals. For variety, the fractions in this visualization reflect frequencies rather than wavelengths, but the latter yields an equivalent result. (**A**) Interference pattern for the interval 60/80 = 3/4 (fourth). This interval is perceived as pure, creating constant sound levels. (**B**) Interference pattern for the interval 80/81 [syntonic comma (*6*)]. Here, the constructive and destructive interference between the two waves leads to a changing amplitude of the interference wave, which is experienced as wavering sound or beat pattern. The interference amplitude alternates between zero and the sum of the two individual amplitudes. Middle row: frequencies 1/60 (**C**), 1/80 (**D**), and 1/81 (**E**). They look almost the same, but the left and center frequencies are added to create the above interference pattern, whereas the center and the right frequencies are added to create the below interference pattern, which is very different.

Slight deviations from pure intonation quickly result in impure intonation, which creates beat patterns. Whereas a pure interval may come through vocally as an assertive “Ta-Daaaa!”, an impure interval will waver, resounding more like some kind of “Wowowowow…” This phenomenon is graphically visualized with waves and interferences in Fig. 3.

Pure and impure intonation behaves differently all the way from the instrument to the human ear. When sound is created on an instrument, pure intonation results in constant sound, whereas impure intonation creates beat patterns. Later, when traveling through the air, pure intonation results in sound waves with many joint nodes, whereas impure intonation results in wavering sound. Last, in the human ear, the sound is perceived through the cochlea, where it makes the ear’s basilar membrane vibrate. There, sound creates resonant wave patterns, and these are orderly if the sound is harmonic but wavering otherwise. Thus, the phenomenon that initially took place on the instrument’s strings and sound box is transferred to the human ear, where it is eventually enhanced through the interaction of sensory and nerve cells.

Opting for pure intonation has some noteworthy limitations. In particular, certain sequences of pure intervals can cause the performer to drift out of tune. This phenomenon was briefly mentioned in the Introduction and may seem somewhat paradoxical. However, it is very common. The example initially mentioned is the sequence “do-so-re-fa-do.” In this sequence, the performer goes a fifth up, a fourth down, a minor third up, and another fourth down. However, if all intervals are pure, the sequence returns to the initial point slightly out of tune. Mathematically, this can be stated as follows: 2/3 × 4/3 × 5/6 × 4/3 = 80/81 ≠ 1. Thus, when going a fifth and a minor third up and two fourths down, one returns slightly out of tune, namely, by a tiny fraction of 80/81, also known as the “Pythagorean” or “syntonic comma.” This tiny difference is perceived as impure, as already established in Fig. 3.

**Fig. 4.**
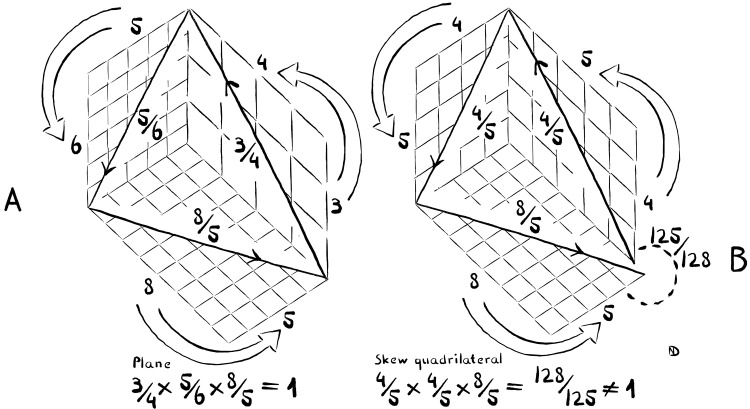
Geometrical representation of the difference between perfect and imperfect tuning. (**A**) Perfect tuning means that all transitions between tones harmonize. In the example shown here, the performer goes down a fourth and a minor third and up a minor sixth, returning to the exact same pitch. Geometrically, each transition between tones can be represented as a slope connecting two axes in a coordinate system. With perfect tuning, the slopes are always fractions of small integers (1/2, 2/3, 3/4, 4/5, 3/5, 5/6, 5/7, and 5/8), and they create an entirely flat shape. In the illustration, one can see the resulting shape rendered as a plane that cuts through all axes. (**B**) Not all sequences of tones can be played in perfect harmony. In this example, the performer goes down two fourths and up a minor sixth. This does not lead back to the exact same pitch. Instead, there is a slight deviation. As seen in the illustration, the shape that is created is no longer flat—the last line sticks out of the plane. During vocal performance and on wind instruments, this geometry requires the performer to slightly swerve, bending the melodic spaces to avoid drifting out of tune.

Sequences of tones that cause the performer to drift out of tune turn out to be relevant in the study of ancient Greek and Roman music. Consider performing such a sequence on a lyre. The problem emerges that one cannot tune the lyre in such a way as to allow for pure intonation throughout. Let us agree on the following two terms: If a sequence of tones can be performed with pure intervals throughout, without ever drifting out of tune, the resulting tuning shall be referred to as “perfect tuning.” In contrast, “imperfect tuning” occurs when this is impossible. Figure 4 visualizes the distinction between perfect and imperfect tuning graphically.

The main idea of Fig. 4 is to compare perfect and imperfect tuning through sequences of tones that return to the initial tone. These sequences can be referred to as “loops.” The visualization demonstrates that a loop of three intervals that can be tuned perfectly can be represented as a flat plane in Euclidean space. On the other hand, a loop that does not allow for perfect tuning must be represented as a curved surface (or as a flat surface in curved, non-Euclidean space).

This idea of a “curved melodic geometry” returns in musical practice. As already discussed, the absence of perfect tuning means that there are impure intervals. On an instrument such as the lyre, this results in wavering sound. However, other instruments, especially the auloi, can boast freedoms in interpretation that the lyre does not have. The same applies to unaccompanied voice. Singers often adopt microtonal adjustments, in more elaborate forms also known as melismas. With melismas, one glides up or down with the voice or swerves in more complex ways. These techniques are also referred to as portamento, vibrato, or pitch bending. They bend the melodic space and can be very valuable, especially if one would drift out of tune without them.

Another solution to bypass syntonic commas is to opt for tempered tuning, as found today in pianos. Rather than playing the intervals in their pure form as they are in the fractions, all intervals are then slightly impure. This allows for simplified math of the kind 5 + 3 − 2 × 4 = 0. In this example, going up a fifth (5) and a third (3) and down two fourths (4) comes back to the same initial position (0). This simplified math is only applicable because the intervals are tempered rather than pure.

Tempering introduces small inaccuracies everywhere, so they do not accumulate but are distributed over the scale. However, with tempering, the performer is always slightly off-pitch. Mathematically, we have a space that lacks precision, akin to a pixelated image. On a piano, this lack of precision can hardly be avoided. Yet, a piano is not a lyre. It is an instrument with hard strings and hammers. Because of this design, some pianists put their beloved instrument into the category of percussion instruments where it stands next to orchestral bells and other metallophones. On a piano, even octaves are played off-pitch. The hard piano strings have an acoustic timbre that forces piano tuners to slightly stretch the octaves. This stretching is an order of magnitude smaller than the swerves necessary in ancient Greek and Roman vocal music. At the same time, no such corrections are required in ancient compositions for lyres, which, with their soft strings, have an unmistakably softer, sweeter timbre.

### What can be observed in ancient Greek and Roman compositions?

The mathematics described above can be applied to study all compositions that have survived from Greek and Roman antiquity, which is what the present article reports on. There are a total of 61 musical fragments, written partly in instrumental and partly in vocal notation [systematically collected and transcribed by Pöhlmann and West (*2*)].

**Fig. 5.**
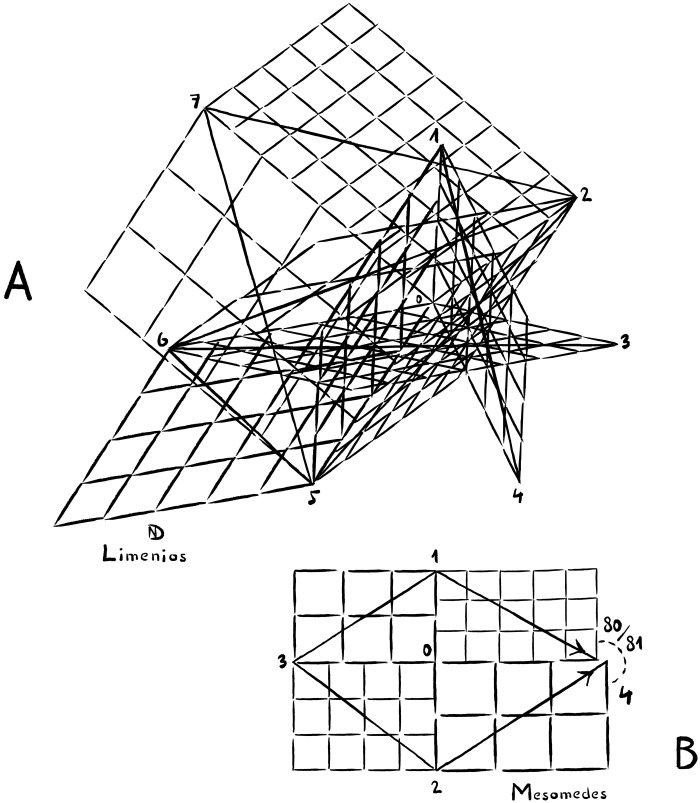
Tuning schemes for two compositions, one in instrumental and one in vocal notation. (**A**) The paean of Limenios, Delphi, reveals perfect tunability in spite of the complex arrangement of intervals. The visual shown here is an axonometric of the seven-dimensional melodic space of Limenios’s seven-stringed composition. All vertices numbered 1 through 7 can be solved accurately, which makes for perfect tuning in this instrumental piece. The lengths of the line segments 0-1 to 0-7 represent the exact wavelength of the tones 1 to 7. (**B**) In contrast, Mesomedes’s “Invocation of the Muse” reveals a tuning conflict right from the start. The sequences 3-1-4 and 3-2-4 result in a syntonic comma. Shown here is an axonometric representation of the four-dimensional melodic space spanned by the first four intervals. This particular piece of vocal music made history for having sparked the birth of modern Opera during the Italian Renaissance.

Stunningly, the mathematical analysis discussed here reveals that ancient Greek and Roman music that has been written in instrumental notation can be purely intonated, not requiring microtonal adjustments or tempering (for the mathematical proofs, see the Supplementary Materials). Composers who wrote their work down in instrumental notation used only combinations of intervals that they were able to tune perfectly on their instruments. Different compositions may require different tuning. However, each composition is always performable in one go, without adjusting the tuning of the instrument while facilitating pure intonation throughout. We have previously referred to this as “perfect tuning.” The choice of perfect tuning in instrumental compositions did require remarkable restrictions, yet it also gave ancient music a sense of natural, pristine beauty. Figure 5A illustrates this type of composition with the Paean of Limenios, 128 BCE, Part 1. Its melodic lines are perfectly straight.

Furthermore, the same mathematical analysis of ancient Greek and Roman music reveals a difference between instrumental and vocal music. Many compositions written in ancient vocal notation do not allow for perfect tuning (for the mathematical proofs, see the Supplementary Materials). This suggests that, in the case of music that was not accompanied by lyres, ancient performers did adopt microtonal adjustments to enrich tonal structure beyond the limits of perfect tuning. Figure 5B illustrates this type of composition with the Invocation of the Muse by Mesomedes. Its melodic lines must be gently curved.

Together, these two observations make perfect sense. Instruments with strings of fixed length (such as lyres) do not allow for microtonal adjustments, and none were required. On the other hand, vocal music does allow for microtonal adjustments, and they are proven to be necessary in the extant musical compositions. Thus, it makes sense that only pieces in instrumental notation allow for perfect tuning because microtonal adjustments are not possible, whereas pieces in vocal notation require microtonal adjustments, which are only possible in this latter case. The observations made through mathematical analysis are entirely reasonable and appear obvious once recognized.

## DISCUSSION

The present analysis reveals the acoustic preferences of composers and audiences during a significant historical period. Were these preferences unique or common?

Pure intonation for an entire genre of music is rather unique to ancient Greece and Rome. By comparison, ancient Mesopotamian music from the second millennium BCE does not allow for perfect tuning. The few fragments that have survived from the ancient city of Ugarit show unavoidable tuning conflicts (*3*).

Today, European and Mediterranean musical styles also require tempering or microtonal adjustments. Specifically, music around the Mediterranean makes abundant use of melismas, as attested, for example, in Byzantine and Arabic music. Likewise, European classical music typically uses tempered musical scales, in which hardly any intervals are pure. Turning to another example, some of the greatest musical hits of the past 50 years allow for perfect tuning, whereas others do not. The cases that allow for perfect tuning may be a chance encounter as this music is not performed on instruments that work best with perfect tuning.

**Fig. 6.**
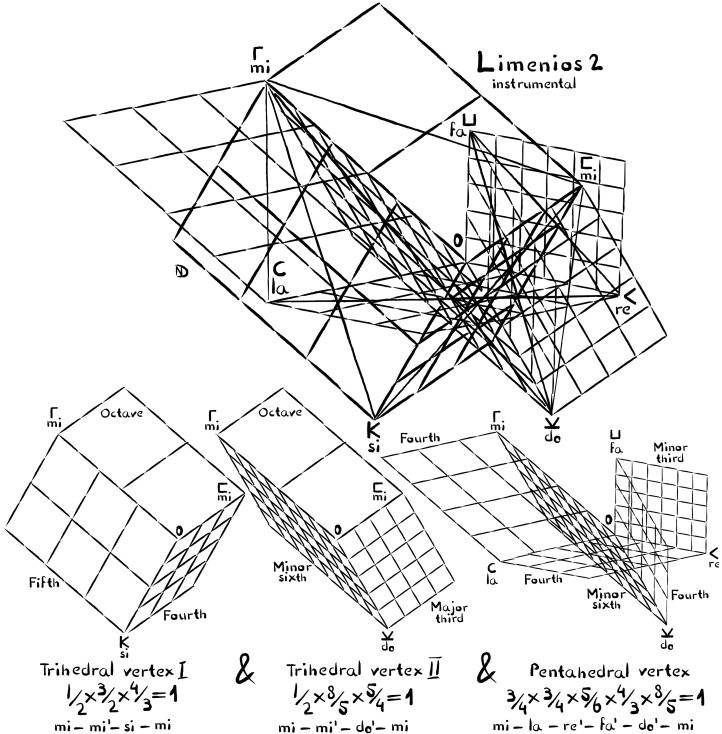
Axonometric of the seven-dimensional melodic space of the second part of Limenios’s composition. Evidently, the composer decided to make his music appealing not by featuring a higher number of tones but by surprising the audiences with a different tuning scheme, perhaps to be performed on a different seven-stringed instrument with matching resonance spectrum. The axonometric above shows the relationships between all seven tones. The exact pitch of each tone is determined by two to four intervals. This makes the melodic space of this piece an over-determined mathematical object, and yet all vertices can be solved accurately, resulting once again in perfect tuning. Below are the two constituent facet-adjacent trihedrals (**left** and **middle**), as well as the pentahedral vertex (**right**) that are combined to generate the scale. Coming up with such an arrangement requires unique musical skill, making this a virtuoso composition worth chiseling on temple walls.

Music that allows for perfect tuning is not easy to compose, and it can impose a strict economy of means on the composers. This is certainly true in the case of ancient Greek and Roman music. Composers who wrote pieces in instrumental notation mostly limited themselves to only seven distinct tones. Acoustic sophistication was achieved not by adding more tones but by recombining a small number of tones in different, unexpected ways. This choice is visualized with the second part of Limenios’s paean, shown in Fig. 6.

**Fig. 7.**
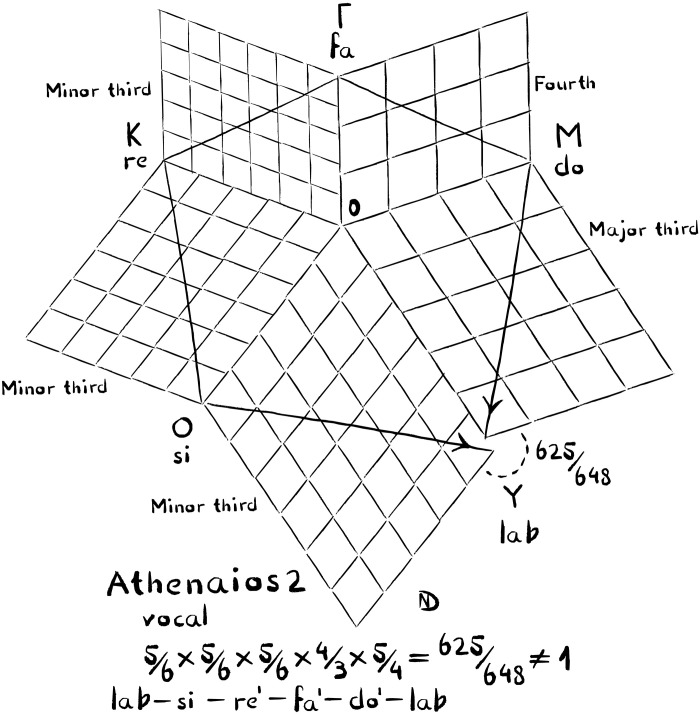
Athenaios’s paean is a vocal counterpart to Limenios’s instrumental composition. Unlike Limenios’s composition, which is perfectly tunable, Athenaios’s paean leads into a tuning conflict. When tone Y is reached via M, it is reached roughly a quarter tone higher. Right at this moment, the lyrics speak of how the music of stringed and wind instruments blends. The illustration shown here is an axonometric of the five dimensions of the melodic space that lead into the tuning conflict. The tone names are given with solfège names as well as in the ancient notation to ensure clarity. The wavelengths are the length of the axes 0-Γ to 0-Y. However, if the intervals are pure, Y must have changing wavelengths with roughly a quarter tone deviation.

Ancient instrumental music is impressive through its harmonic integrity. Vocal music is equally artful, especially because it can demand high skill in modulating tones. This can be demonstrated, for example, with the vocal counterpart of Limenios’s paean. Composed by Athenaios, this vocal piece leads into a tuning conflict of roughly the size of a quarter tone. Figure 7 visualizes graphically how the melody swerves away from a perfect tuning scheme. The performer had to somehow handle the deviation. Did he or she perform the intervals impure, or opt instead for gently swerving with the voice? Exactly this choice made the music different from lyre performances and certainly very moving.

**Fig. 8.**
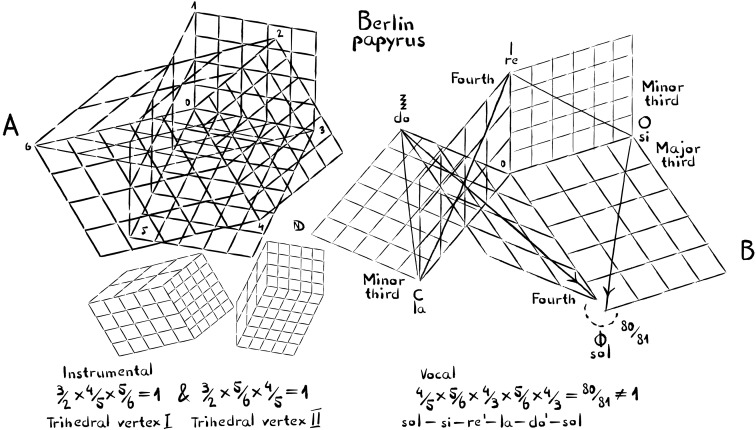
The “Berlin Papyrus” contains a vocal and multiple instrumental compositions, which have been interpreted to belong together. As in the case of the paeans of Delphi, the instrumental compositions allow for perfect tuning, whereas the vocal composition leads into a tuning conflict. This is quite remarkable, given that the Berlin Papyrus compositions are dated from the Roman period, three centuries after the paeans of Delphi. (**A**) The instrumental compositions feature a recurring tuning scheme with two trihedral vertices, rotated by the fourth 6-3. One trihedral vertex composed of fifth, major third, and minor third is shown separately (bottom left). The other, composed of fifth, minor third, and major third, is shown separately (bottom right). This type of tuning may have been frequent. Ptolemy referred to it as “intense diatonic.” (**B**) Axonometric of five tones from the vocal composition, showcasing how they lead up into a syntonic comma at Φ. The intervals are the same as those mentioned when tuning compromises were explained in the Introduction and the Results sections. \

The case of Limenios and Athenaios demonstrates the uniqueness of ancient Greek music. No other musical tradition is known to so clearly distinguish between perfect and imperfect tuning. However, this distinction remained foundational for both Greek and Roman music. Three centuries after Athenaios and Limenios, a very different set of vocal and instrumental compositions still lived from the same distinction between perfect and imperfect tuning. In fact, nothing changed. Here, too, the instrumental pieces were perfectly tunable, whereas the vocal piece swerved away from linearity (Fig. 8).

Is the same artistic touch also found in other ancient Greek and Roman arts? This article’s Introduction mentioned temple architecture, which appears simple and is yet highly sophisticated, with straight lines that are actually gently curved. In addition, gently curved geometries return in ancient Greek and Roman philosophy—atomism made a direct connection between gentle curves and free will.

Atomism viewed the universe as composed of tiny atomic elements. They were too small to see and too numerous to count, but they nevertheless made up our entire material world. In this context, some philosophers recognized that combining and recombining atoms in linear ways must have limits. In consequence, they imagined that some kind of nonlinearity or “swerving” must be necessary to support additional complexity. This is attested, for example, in the work of Lucretius.

In his poem “On the Nature of Things [De Rerum Natura],” Lucretius discussed the concept of the “swerve” or “inclination” [clinamen] of atoms—an infinitesimally small deviation in their linear motion. This deviation was crucial as it introduced an element of nonlinearity and unpredictability in a universe primarily governed by linearity. Lucretius explained this idea in Book II of his poem, where he posited that without swerving, all motion would be completely linear and everything would fall straight down through the void like raindrops. Here’s a brief portion of the text discussing this concept, translated into English:

“The atoms move by themselves... But when they are traveling straight down through empty space by their own weight, at quite indeterminate times and places they swerve ever so little from their course. [Without swerving] bodies would never be able […] to produce the various motions by which nature carries its processes. […] Furthermore, […] without swerving […] there would be no free will in living beings all over the earth.” (*4*)

**Fig. 9.**
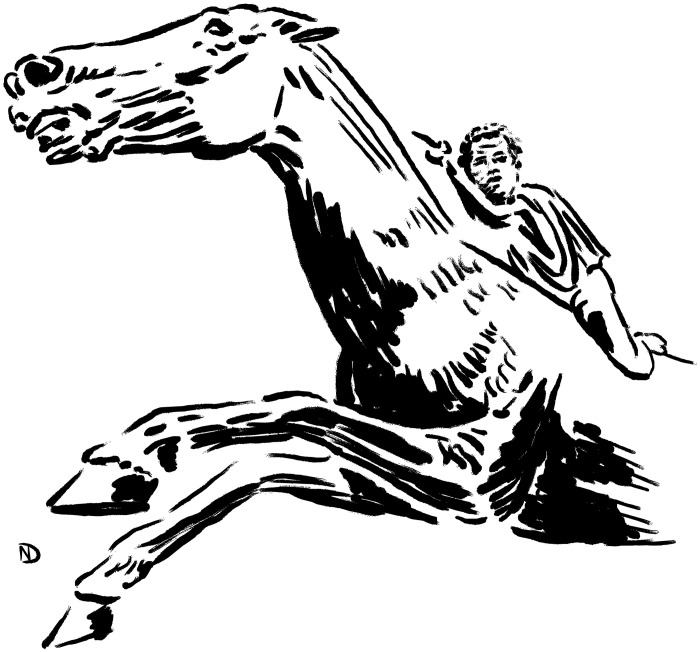
The Jockey of Artemision visualizes a horse race in antiquity. Lucretius held that the force to accelerate forward and win a race originates in the slight swerving behavior of atoms, which initiates complex patterns of behavior.

Clearly, Lucretius considered that nonlinearity began with small swerves and individual inclinations. This also comes to bear in Lucretius’s verses about horse races, where the horses accelerate, although only gradually. It takes time for the individual inclinations to spark a larger, physically observable change. Here are Lucretius’s own words:

“Do you not see when the cells are thrown open that nevertheless the horses cannot burst forward as fast as the mind itself craves?” (*4*) (Fig. 9)

**Fig. 10.**
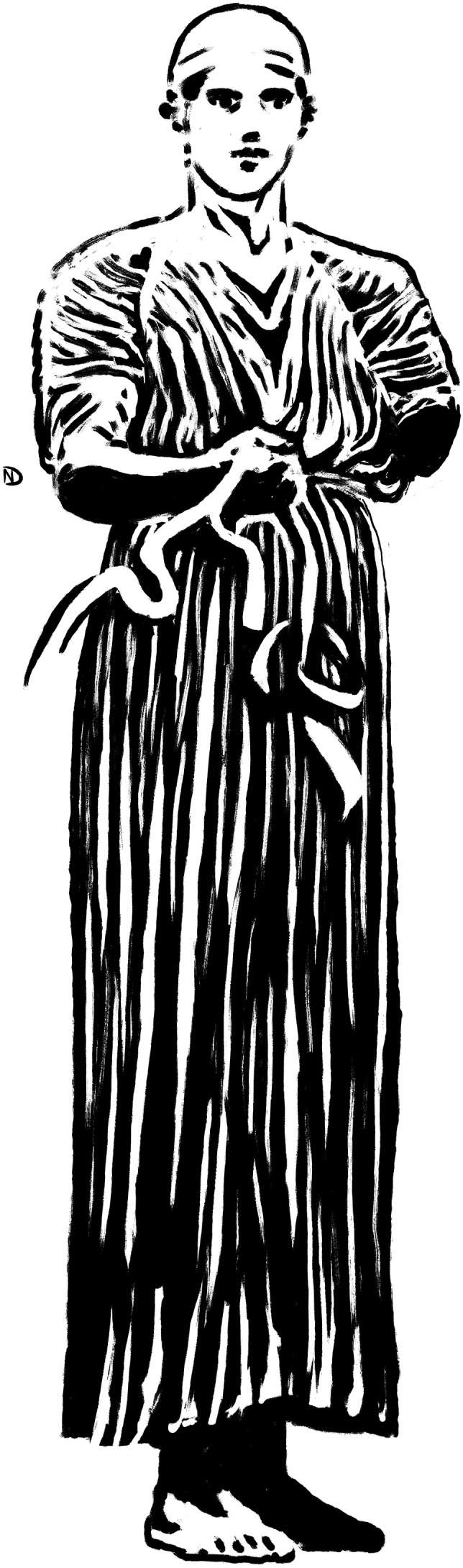
The Charioteer of Delphi. Symbolizing inner peace, the charioteer accepts his victory as if it were preordained, embodying serene composure. Positioned alongside the Jockey of Artemision shown in Fig. 9, these images exemplify the dual modes of Greek art. This duality is paralleled in the musical distinctions found in the meticulously composed pieces for the lyre, demonstrating pure and structured harmony, versus the vivid, free-spirited compositions for auloi. Dating back to around 470 BCE, the charioteer is an exquisite bronze sculpture commemorating a chariot race victory at the Pythian Games.

The swerving described by Lucretius has a matching counterpart in ancient musical compositions. While compositions written in instrumental notation remained limited in tonal complexity and perfect in their harmony, vocal compositions added musical complexity even at the cost of breaking limitations—they forced the performer to swerve. Graphically, this swerving can be represented with slightly curved geometries, as already shown in Fig. 4B. In addition, it is reflected in the postures of the performers who accentuated musical nuances, giving them playful expression, as shown earlier in Fig. 2B. Ancient Greek music, art, and philosophy often lived from the juxtaposition of such free-spirited exuberance on one hand (as shown in Figs. 2B and 9) and farsighted, unerring perfection on the other (as seen in Figs. 2A and 10). The same distinction reverberates through history as a distinction between reasoning and sentimentality, reflection and spontaneity, or perhaps simply linearity and nonlinearity.

### What other, present-day perspectives can be explored in ancient music?

This article has engaged with ancient Greek and Roman music through a mathematical and acoustical analysis while also exploring philosophical, historical, and artistic perspectives. For further exploration of an additional, present-day perspective, visit the online project page associated with this article. There, you will find access to the Grombot family—a collection of Ancient Greek and Roman Music Bots, trained exclusively on ancient musical material. They use a robot’s setup to create new musical compositions in ancient styles. See Box 1 for terms used throughout the article.

Box 1. **Glossary.**

A note: A symbol used in musical notation to represent a sound with a specific pitch. Notes are fundamental units in musical composition, representing the sounds produced by instruments or the human voice in written form.

A tone: The actual sound produced by a musical instrument or the human voice, characterized by its pitch. The tone includes the fundamental frequency or wavelength and a series of overtones, which are higher frequencies that typically occur at whole number multiples of the fundamental frequency. These overtones contribute to the distinctive quality or timbre of the sound.

An interval: In music, an interval refers to the difference in pitch between two tones. It is the basis for harmony and melody in music. Intervals are classified by their size (e.g., third, fourth, fifth, and octave).

A pure interval: A pure interval occurs when the frequencies of its two constituent tones have a simple mathematical ratio, such as 1/2 for an octave or 2/3 for a perfect fifth. This results in a consonant sound where the waveforms align, minimizing auditory roughness and maximizing harmonic agreement.

A loop: A sequence of musical intervals that starts and ends on the same tone, creating a complete cycle.

Perfect tuning: Perfect tuning is a tuning system in which all intervals utilized in a composition are tuned to have pure ratios, eliminating any beating or dissonance that occurs when intervals are slightly out of tune. In perfect tuning, the frequencies of the notes are fixed and do not vary. This arrangement ensures that compositions played in this tuning system maintain their intended harmonic clarity and integrity from start to finish. In perfect tuning, all loops return to the exact same pitch.

Microtonal adjustments: Microtonal adjustments involve altering the pitch of a tone.

Portamento: Portamento is a musical expression technique where the pitch slides continuously from one note to another, often used in singing to enhance expressiveness by connecting two tones seamlessly.

## MATERIALS AND METHODS

The mathematical analysis of the musical fragments that remain from Antiquity is represented in the Supplementary Materials.

## Supplementary Materials

### This PDF file includes:

Mathematical analysis Instrumental pieces Sample vocal pieces Comments on source material and its context
