# Supplementary material for: Human touch? Acoustical analysis of ancient music reconstructs tuning and intonation, elucidating aspects of human behavior
Source: Sci Adv. 2025 May 7;11(19):eadv3101. doi: 10.1126/sciadv.adv3101 (PMC12057680; doi:10.1126/sciadv.adv3101)
Supplement: Supplementary file 1 — Mathematical analysis Instrumental pieces Sample vocal pieces Comments on source material and its context [file sciadv.adv3101_sm.pdf]

Science Advances
AAAS

Supplementary Materials for

Human touch? Acoustical analysis of ancient music reconstructs tuning and intonation, elucidating aspects of human behavior

Dan C. Baciu

Corresponding author: Dan C. Baciu, dan.baciu@fh-muenster.de

Sci. Adv. **11**, eadv3101 (2025)
DOI: 10.1126/sciadv.adv3101

This PDF file includes:

Mathematical analysis
Instrumental pieces
Sample vocal pieces
Comments on source material and its context

SUPPLEMENTARY MATERIAL

Mathematical analysis

To support other researchers in replicating the present research as well as to support composers in understanding the tonal structure of some of ancient Greek pieces, the present section puts to paper the most relevant mathematical considerations for each composition. All instrumental compositions are featured together with the vocal compositions that contain tuning conflicts.

Readers are encouraged to double-check the present evaluation as well as analyze the remaining vocal fragments independently, to test or replicate the present observations.

The numbering of the pieces that are followed here is taken from **P**öhlmann & **W**est (2001). The pieces are referred to as "PW" followed by the fragment number given in that publication.

Pöhlmann & West (2001) split the Paean of Limenios into ten sections, following the ancient engraving. Here, these sections are numbered following that publication. References to page numbers are included for convenience.

The mathematical analysis is straightforward. For each instrumental piece, all intervals are listed that are at least the size of a minor third, together with their value as a fraction. In all instrumental pieces, perfect tuning can be obtained, and there are no tuning conflicts. To avoid creating artifacts, it is recommended not to tune intervals from reconstructions newly proposed by modern scholars, and not to tune over lacunas. In this way, all tuned intervals are properly attested in the originals.

For each vocal piece, the present section is limited to listing the intervals that lead to tuning conflicts. This saves space and time for the readers.

Western traditions have keyboard and solfège names for tones. The present section utilizes the latter because they are the most widespread tone names today. (Do, Re, Mi, Fa, So, La, Si, Do'.) The ancient Greek notation is found in Pöhlmann & West (2001) as well as in several of the article's illustrations. To avoid any misunderstanding, it is recommended not to merge any two different ancient Greek note symbols to the same Solfège-value.

As mentioned, fractions can be used to denote the tuning. An octave is 1/2. A fifth is 2/3. A fourth is 3/4. Major and minor thirds are 4/5 and 5/6, respectively. Major and minor sixths are 3/5 and 5/8, respectively. Additionally, there is a wider form of the fourth, representable as 5/7, which may appear in the Paean of Limenios, Part 1.

In this notation, a fifth up is 2/3, while a fifth down is 3/2. Please note that tuning conflicts arise regardless of whether an interval is played up or down, so this notation style needs not be followed.

Some intervals are played both up and down. This is not always recorded in the following analysis, as it has no relevance for the tuning.

Compositions without tuning conflicts can be represented as shapes with the following characteristics: for a musical scale with n tones, all intervals equal to a minor third or greater can be represented as slopes with fractions composed of integers equal or smaller than 8, and if all slopes are connected, they create an up to an $n - 1$ dimensional shape that is flat, not curved. By contrast, compositions with tuning conflicts require slight curvatures. This means that scales with tuning conflicts can be represented as curved spaces. Because the curvature of a space is detected

by studying loops in it, it follows that for a composition to lead into tuning conflicts, it is necessary that there is the possibility of a loop, which goes up the scale in one sequence of pure intervals and comes down in another, returning to the same tone.

In the instrumental pieces that survive from antiquity, harmonic loops are often avoided, and where they occur, they do not lead into tuning conflicts. Note that all loops in instrumental pieces can be written with a sequence of multiplications of fractions made up of small integers that equals exactly 1. This means that the loop can be performed with pure intervals, returning to the exact same tone.

The same is not the case for loops in vocal pieces, which often do not return to the exact same tone. This means that there is a tuning conflict. Either the space is curved, or the loop does not return to the same point, necessitating microtonal adjustments. Arithmetically, the sequence of multiplication does not equal exactly 1.

As can be seen, the instrumental pieces tend to avoid loops, yet they feature at least eleven, all of which can be tuned perfectly. No exception to perfect tuning has been found. By contrast, the vocal pieces feature at least thirteen loops, which cannot be tuned perfectly.

While intervals smaller than a minor third are not recorded in the following analysis, they can mostly be reconstructed based on the larger, more clearly perceptible intervals. Smaller intervals of tones, half-tones, and quarter-tones also have clean fractions in instrumental music. Examples are 8/9, 9/10, 14/15, 15/16, 20/21. Similar fractions have attracted the attention of ancient theoreticians. The intervals of seven or nine tones, which are commonly perceived as dissonant and are therefore not used for tuning, result in fractions with similar integers.

<u>*Instrumental pieces*</u>

PW 15. Instrumental and vocal.

The instrumental intervals played are: Fa’-Do’ 4/3; La-Do’ 5/6; Si-Mi 3/2; Mi-Si_ 4/3; Si_-Si 1/2; Mi-La 3/4.

Loop: Si-Mi-Si_ = 3/2 × 4/3 × 2/1 = 1.

No tuning conflict arises among the instrumental intervals. Seven distinct instrumental tones.

PW 16. Instrumental and vocal.

This fragment follows PW 15. It is added here for the sake of completeness as it contains instrumental notation, although no interval of a minor third or above. Obviously, there are no tonal loops and no tuning conflict. There are fewer than seven distinct tones.

Limenios, Delphi paean, part 1 (PW 21.1—p. 74f) Instrumental.

Fa’-Re’ 5/6; Re’-La 4/3; Re’-Si♭ 5/4; Si♭-Fa’ 2/3; Mi’-Si♭ 5/7; So’-Mi♭’ 4/5; (So’-Re’ 3/4 may have been tuned over So’-Mi♭’-Re’).

Loop: Fa’-Re’-Si♭-Fa’ = 5/6 × 4/5 × 3/2 = 1.

A total of seven distinct tones are played. This fits to an instrument such as the lyre chiseled on the same temple wall. No tuning conflict arises. Thirds add up to a fifth. Fourths are very skillfully combined with thirds and fifths in ways that lead to no conflict. Intervals that cannot be purely intonated are not played. Mi’-Si♭ could be a tritone, yet it is more harmonic if played as a 5/7. This would furthermore make the halftone Fa’-Mi’ amount exactly to a sweet-sounding 15/14.

Parts 2, 3, 4, 7, and 8 of this composition feature seven distinct tones. Of the remaining three parts, part 6 features six attested tones, but a tone may be missing in a lacuna. Part 5 features an uncertain number of tones between 6 and 8. For details see explanation in Pt. 5. And Part 10 features eight tones. Therefore the number of tones is mostly seven, with only one certain deviation by a single tone. The slightly higher number of tones in the last part makes sense. Being the last part, audiences expect something extraordinary, and the tuning scheme of this part really is ingenious. The recurrent number of seven tones matches up with depictions of lyres. This may suggest that the ten different parts were played on separate instruments. Such an

arrangement would have let performers benefit from the unique frequency response and resonance characteristic of the instruments, performing a particular scale on an ideally suited instrument. Such a match between instrument and composition was certainly desirable, given that the pieces were performed in public and without acoustic enhancements.

Limenios, Delphi paean, part 2 (PW 21.2—p. 76f) Instrumental.

La-Mi 3/4; Fa’-Re’ 6/5; Mi-Mi’ 1/2; Mi’-Do’ 4/5; Si-Mi 3/2; Mi-Do’ 5/8; Do’-Fa’ 3/4.

Loop: Mi-Mi’-Do’-Mi = 1/2 × 8/5 × 5/4 = 1.

Loop: Mi-Mi’-Si-Mi = 1/2 × 2/3 × 3/4 = 1.

Loop: Mi-La-Re’-Fa’-Do’-Mi = 3/4 × 3/4 × 5/6 × 4/3 × 8/5 = 1.

A total of seven tones are played. As before, this again fits to an instrument such as the lyre chiseled on the same temple wall. Octave, fifth, fourths, thirds, and a minor sixth are used. Three loops emerge, one of which is very elaborate going two consecutive fourths and a minor third up, and returning over a fourth followed by a minor sixth down. No tuning conflict arises.

Limenios, Delphi paean, part 3 (PW 21.3—p. 76f) Instrumental.

Mi’-Do’ 5/4; Mi’-Mi 1/2; Mi-Si 2/3; Mi’-La’ 3/4.

Avoids loop.

A total of seven tones are played. As before, this again fits to an instrument such as the lyre chiseled on the same temple wall. No tuning conflict arises.

Limenios, Delphi paean, part 4 (PW 21.4—p. 78f) Instrumental.

La-Re’ 3/4; Re’-Si 6/5; Mi ♭ ’-So’ 4/5; La-Mi’ 2/3; Si ♭ -Re’ 5/4.

Avoids loop.

A total of seven tones are played. As before, this again fits to an instrument such as the lyre chiseled on the same temple wall. No tuning conflict arises. This part contains also a quarter-tone of 24/25 between major and minor thirds.

Limenios, Delphi paean, part 5 (PW 21.5—p. 78f) Instrumental.

Fa’-Re’ 6/5; Re’-Si 6/5; Mi’-Mi 2/1; Mi-Do’ 5/8; Do’-Mi’ 4/5; Si-Fa 4/3; Fa-La 4/5.

Loop: Mi-Mi’-Do’-Mi = 2/1 × 5/8 × 4/5 = 1.

The composition is well developed. Octave, major and minor thirds are arranged in an ingenious way. No tuning conflict arises.

This piece contains two tones that are not well documented. They do not affect the tunability of the piece, but we are uncertain of the accuracy of the reading. Our inspection of the fragment on site in Delphi casts doubt. Line PW-21.5.20, contains two tones that appear only once, without reappearing anywhere else in this part of the paean. Furthermore, these two tones are located on a part of the stone that has experienced more significant degradation. On close look, we found the two symbols—and L and a C. However, the L was hardly readable. It is missing a clear vertical line, which could not have disappeared due to bad handling of the stone, but must not have been there from the beginning. In addition, the horizontal line is located close to other horizontal lines that do not denote tones. Perhaps this is another tone or no note symbol at all. The C is more likely an actual note symbol, but it quite clearly features straight parts, as a <, which is a frequent tone in this composition, being repeated at least six times in the better readable places. It is true that some occurrences of C are somewhat edgy, but this piece does not feature another C, which makes the occurrence statistically much less likely than a <.

Limenios, Delphi paean, part 6 (PW 21.6—p. 80f) Instrumental.

Re’-Fa’5/6; Fi’-Mi 6/5; Mi-Mi’ 1/2; Mi-La 3/4; La-Mi’ 2/3.

Loop: Mi’-Mi-La-Mi = 2/1 × 3/4 × 2/3 = 1.

A total of six tones are played. Octave and minor thirds are arranged in an uncommon way. No tuning conflict arises. Part 6 and 7 do not have a clear separation, but even if played together, they show no tuning conflict.

Limenios, Delphi paean, part 7 (PW 21.7—p. 80f) Instrumental.

La-Re’ 3/4; La-Mi’ 2/3; So-Si 4/5.

Avoids loop.

A total of seven tones are played. No tuning conflict arises.

Limenios, Delphi paean, part 8 (PW 21.8—p. 80f) Instrumental.

Do'-Fa' 3/4; Fi'-Re' 5/4; Fa'-Re' 6/5.

Avoids loop.

A total of seven tones are played. No tuning conflict arises.

Limenios, Delphi paean, part 9 (PW 21.9—p. 82f) Instrumental.

Fa'-La' 4/5; Re'-Fa' 5/6; Mi'-Mi 2/1.

Avoids loop.

A total of seven tones are played. No tuning conflict arises.

Limenios, Delphi paean, part 10 (PW 21.10—p. 82f) Instrumental.

Fa'-Re' 6/5; Si ♭ -Re' 4/5; Re'-La 3/4; Si ♭ -Fa' 2/3; So'-Mi ♭ ' 5/4; Si ♭ -So 6/5.

Loop: Fa'-Re'-Si ♭ -Fa' = 6/5 × 5/4 × 2/3 = 1.

No tuning conflict arises. A total of eight tones are played, perhaps to impress audiences with a slightly different tonal arrangement, featuring an extra tone. This last segment also features an ingenious tuning scheme, completing this beautiful paean. The reading that Pöhlmann & West (2001) contains a note marked as "uncertain note symbol." The note would bring into the melody an otherwise uncommon tritone (here as Mi ♭ '-La, reconstructable as 45/64 under the assumption that the octave is pure). While a tritone is an interval that is mostly perceived as dissonant, its use in this composition would not lead to a tuning conflict. However, it should be said that on inspection in Delphi, the note symbol was found to be entirely absent, being located on the damaged border of the stone towards a lacuna.

Instrumental composition (PW 32) Instrumental.

Fa-Si ♭ 3/4; Si ♭ -Re' 4/5; Re'-Fa' 5/6; Do'-So 4/3; Fa-Fa' 1/2.

Loop if two compositions played together: Fa-Fa'-Re'-Si ♭ -Fa = 1/2 × 6/5 × 5/4 × 4/3 = 1.

These are two pieces, written in one, but stretching over the line break of the column. If they are played separately, they have likely 7 and 6 or 8 and 5 distinct tones, depending on where the separation between the two pieces is made, following the music or the line break in the column. They have different tones and may not have been played together, but even if they were, no tuning conflict arises.

Instrumental composition (PW 33) Instrumental.

Re-Fa 5/6; Mi-So 5/6; Re-So 3/4.

No loop.

No tuning conflict arises. Four distinct tones.

Instrumental composition (PW 34) Instrumental.

Re-Fa 5/6; Mi-So 5/6; Re-So 3/4.

No loop.

No tuning conflict arises. Four distinct tones.

Instrumental composition (PW 35) Instrumental.

Re-So 3/4; La-Fa 4/5; Fa-Re 5/6; Mi-So 5/6.

No loop.

No tuning conflict arises. Four pieces of five, five, six, and five distinct tones.

Instrumental composition (PW 36) Instrumental.

This is likely an exercise of playing a list of tones up and another down, not a musical composition in the common sense of the word. There is no interval of or above 5/6 and no loop. No tuning conflict arises.

Instrumental composition (PW 37) Instrumental.

La-Re 3/2; So-Mi 6/5; Re-Fa 5/6; Fa-La 4/5.

Loop: La-Re-Fa-La = 3/2 × 5/6 × 4/5 = 1.

Thirds add to fifth. No tuning conflict arises. These are four pieces of six, six, five, and five tones.

Instrumental composition (PW 51).

So#-Do 4/5; Do'-La 6/5; So#-Re' 2/3; Mi'-La 3/2 (Fragment 51.13); Si-Re' 5/6; Re'-Fa 5/6 (additionally from 51.14, if played together); La'-Mi' 4/3 (additionally from 51.15, if played together).

No loop.

No tuning conflict arises. Thirds and fifths are beautifully arranged. These are three lines, each appearing to start with different material. The number of distinct tones is seven, nine, and six or seven.

Instrumental composition (PW 52).

Fa-La 4/5; Re-So 3/4; So-Mi 5/6; Do-Mi 4/5; Re-La 2/3; Fa-Re 5/6.

Loop: Fa-La-Re-Fa = 4/5 × 3/2 × 5/6 = 1.

No tuning conflict arises. Thirds and fifth and fourth are beautifully arranged. Each line starts with different material, having a total of seven, six, and five tones.

Instrumental composition (PW 61).

Re'-La' 2/3; La'-Fa' 5/6; Re'-So' 3/4; Mi'-So' 5/6; Si ♭ -Re' 4/5.

No loop.

No tuning conflict arises. Fourteen different notes are employed.

PW 3. Instrumental and vocal.

Fa-Si.

No loop.

This early fragment is added here for the sake of completeness, given that it contains one instrumental interval. Obviously, this interval can be purely intonated and no tuning conflict can arise.

PW 11. Instrumental and vocal.

This fragment is added here for the sake of completeness as it contains instrumental notation, although no interval of a minor third or above. Obviously, there is no loop and no tuning conflict.

Sample vocal pieces

Athenaios, Delphi paean, part 2 (PW 20.2). Vocal, with tuning conflict.

A tuning conflict arises in Fa'-Re'-Si-La♭-Do'-Fa'.

Mathematically $6/5 \times 6/5 \times 6/5 \times 4/5 \times 3/4 = 648/625 \neq 1$

This vocal piece can be interpreted as a counterpart to Limenios's Delphi paean, i.e. the instrumental composition PW 21, which has no tuning conflicts. Unlike Limenios's Delphi paean, this piece does not have any notation to distinguish between different sections. We nevertheless look at part 2 separately, and it has a tuning conflict even if the part is played independently. However, we would like to suggest that the markings used in Limenios's Delphi paean may have been used to indicate a change of instrument, while larger portions of this present paean could have been played on one instrument. Given that tuning conflicts arise anyway in vocal music, it may have made it less necessary for the performer to change instruments.

Mesomedes, invocation of the muse (PW 24). Vocal, with tuning conflict.

A tuning conflict arises in the first few notes, La-Mi', Mi'-So, La-Re', Re'-So. These can be chained to the loop La-Mi'-So-Re'-La, which starts with a La and returns to a different La, not identical to the initial one.

Mathematically $2/3 \times 5/3 \times 2/3 \times 4/3 = 80/81 \neq 1$

This vocal piece has sometimes been interpreted as similar to the instrumental composition PW 23, which has no tuning conflict.

Mesomedes, hymn to the sun (PW 27). Vocal, with tuning conflict.

A tuning conflict arises with the intervals So-Si♭ 5/6; Si♭-Re’ 4/5; Re’-La 3/4; La-Do’ 5/6; Do’-So 3/4. A fifth and a minor third up are not equal to two fourths down. Mathematically $5/6 \times 4/5 \times 4/3 \times 5/6 \times 4/3 = 5/9 \times 16/9 = 80/81 \neq 1$

This vocal piece is in the series of compositions of Mesomedes.

Mesomedes, hymn to Nemesis (PW 28). Vocal, with tuning conflicts.

A tuning conflict arises with the intervals Do’-So’ 2/3; So’-Re’ 4/3; Re’-Fa’5/6; Fa’-Do’ 4/3. A fifth and a minor third up are not equal to two fourths down. Mathematically $2/3 \times 4/3 \times 5/6 \times 4/3 = 5/9 \times 16/9 = 80/81 = 80/81 \neq 1$

A second conflict arises as in the previous piece with the intervals So-Si♭ 5/6; Si♭-Re’ 4/5; Re’-La 3/4; La-Do’ 5/6; Do’-So 3/4. A fifth and a minor third up are not equal to two fourths down. Mathematically $5/6 \times 4/5 \times 4/3 \times 5/6 \times 4/3 = 5/9 \times 16/9 = 80/81 = 80/81 \neq 1$

This vocal piece has two tuning conflicts. It is the last in the series of compositions of Mesomedes, the majority of which are listed here with tuning conflicts.

PW 38. Vocal, with tuning conflict.

A tuning conflict arises with the intervals Do’-So’ 2/3; So’-Re’ 4/3; Re’-Fa’5/6; Fa’-Do’ 4/3. A fifth and a minor third up are not equal to two fourths down. Mathematically $2/3 \times 4/3 \times 5/6 \times 4/3 = 5/9 \times 16/9 = 80/81 = 80/81 \neq 1$

PW 39. Vocal, with tuning conflicts.

A tuning conflict arises with the intervals La-Do’ 5/6; Do’-Mi’ 4/5; Mi’-Si 4/3; Si-Re’ 5/6; Re’-LA 4/3. A major and two minor thirds up are not equal to two fourths down. Mathematically $5/6 \times 4/5 \times 4/3 \times 5/6 \times 4/3 = 5/9 \times 16/9 = 80/81 \neq 1$

Another tuning conflict arises with the intervals So#-Do’ 4/5; Do’-Mi’ 4/5; Mi’-Si’ 3/4; Si-So# 5/6. Two major thirds up are not equal to a fourth and a minor third down. Mathematically $4/5 \times 4/5 \times 4/3 \times 6/5 = 16/25 \times 24/15 = 128/125 \neq 1$ (off by 1/4 tone)

Another tuning conflict arises with the intervals Mi-So# 4/5; So#-Do' 4/5: Do'-La 5/6; La-Mi 3/4. As just before, two major thirds are not equal to a minor third and a fourth. $4/5 \times 4/5 \times 4/3 \times 6/5 = 16/25 \times 24/15 = 128/125 \neq 1$ (off by 1/4 tone)

Another tuning conflict arises with the intervals Mi'-So 5/3; So-Mi 6/5; Mi-Do' 5/8; Do'-So# 5/4; So#-Si 5/6; Si-Mi' 3/4. Mathematically $5/3 \times 6/5 \times 5/8 \times 5/4 \times 5/6 \times 3/4 = 2/1 \times 25/48 = 25/24 \neq 1$ (off by 1/4 tone)

Additional tuning conflicts arise from the previous sequence. For example, the sixth in the sequence is also combined from a fourth and a third, which add up to a sixth, which, as we have seen, conflicts with the rest of the sequence.

PW 42. Vocal, with tuning conflicts.

A tuning conflict arises with the intervals Re'-So' 3/4; So'-Mi' 5/6; Mi'-La' 3/4; La'-Re 3/2. Mathematically $3/4 \times 6/5 \times 3/4 \times 3/2 = 81/80 \neq 1$

Vocal composition (PW 50). Vocal, with tuning conflict.

A tuning conflict arises with the intervals La-Re' 3/4; So-Do' 3/4; La-Do' 5/6; So-Si 4/5; Si-Re 5/6. If the first three are pure, the latter two cannot also be pure. Mathematically $4/3 \times 5/6 \times 4/3 \times 4/5 \times 5/6 = 4/3 \times 4/3 \times 5/6 \times 2/3 = 16/9 \times 10/18 = 80/81 \neq 1$.

This vocal piece has been interpreted by PW as a counterpart to the instrumental compositions PW 51 and PW 52, which have no tuning conflict.

PW 53. Vocal, with tuning conflict.

Fragments 5.2 and 5.4 are taken to belong to the same composition, as suggested by Pöhlmann & West (2001). This makes sense based on lyrics, handwriting, and musical notes employed.

A tuning conflict arises with the intervals Re'-Re 2/1; Re-La 2/3; La-Do' 5/6; Do'-So 4/3; So-Re' 2/3. Mathematically $2/1 \times 2/3 \times 5/6 \times 4/3 \times 2/3 = 80/81 \neq 1$.

If fragment 53.1 is considered to belong to the same composition, as conjectured by PW, an additional tuning conflict arises over Re'-La 4/3.

Comments on source material and its context

Imagine if only the Parthenon had survived from antiquity, and archaeologists discovered its gentle curvature. Would they still conclude that the curvature was designed on purpose? What if several additional temples were discovered, which also had curvature, although these temples were located in different countries and stemmed from different periods? Would this not further attest to the widespread use of curvature? And what if a type of architectural drawing was attested that was used for drawing gentle curves? Would this not further demonstrate that there was an artistic skill or tradition linked to the curvature? Ultimately, evidence from antiquity is limited, and conclusions must be drawn carefully.

In the present case, the source material is limited but good. Some facts are certain beyond any reasonable doubt.

Certainly, there was a widespread association of the lyre with Apollo (attested since the Homeric Hymn of Hermes and in art) and that of wind instruments with Dionysus (attested for example in tragedies and in art). From this association, we can clearly deduce that the two instruments were indeed used for different kinds of music. Lyres played more ordered compositions while auloi allowed for freer melodic arrangements, as evidenced by both images and text that have survived.

In parallel, there existed two, completely different styles of musical notation: instrumental and vocal notation. They were used over multiple centuries, next to each other. Such an occurrence makes only sense if these styles of notation reflected different styles of musical composition or performance. Which could these styles be? The stylistic distinction between music performed on lyres and music performed on auloi is the only fitting candidate. This

distinction is deeply rooted in ancient Greek mythology and symbolism, and it makes sense that such a distinction is also reflected in the existence of two styles of notation. (By comparison, European musical tradition has only one style of notation, but it has two sets of names, keyboard and solfège names, with one being rooted in the history of keyboards while the other stems from vocal traditions.)

Another fact known beyond doubt is that lyres are most frequently shown with an average of seven strings. What could have been the reason for keeping this design? One fact is known with certainty. Lyres were used to perform music in theaters, and this was done without acoustic enhancements. This use of the instruments certainly put a strain, especially on lyres with their limited resonance spectra. Thus, it makes sense to limit oneself to compositions with only a few distinct tones, matching precisely an instrument's resonance spectrum.

Furthermore, it is certain that ancient theoreticians knew how to represent intervals with fractions. Pure intonation comes naturally to many people, but the fact that someone knows how to represent it with fractions makes the familiarity certain beyond doubt. In this context, it should be noted that it makes sense to use pure intonation, especially considering the context of lyre performances in public. In such a context, it is important to make the most of the acoustic properties of sound, opting for pure intervals. It is certain that two strings played in sequence resonated together on a lyre, and with pure intervals, the resulting sound was harmonic and constant, not wavering.

These facts already set clear expectations. Before even looking at ancient compositions, one could expect to find a distinction between music for lyres and music for auloi, with the

former being limited to few tones and purely intonated intervals, while the latter could inspire freedom from such restrictions. The evidence at hand reconfirms exactly this picture.

The Paean of Limenios is an outstanding source with ten different compositions, all of which demonstrate unique skill. They allow for perfect tuning despite the fact that the tuning is overdetermined by the multitude of intervals played during each composition. At the same time, each composition could have been performed on an instrument featuring only seven strings.

Next to Limenios, there is a source dated to the imperial period, three centuries after the paeans of Delphi. With multiple truly beautiful compositions (PW 51.1-3 and 52), this source demonstrates the same attention to tuning, showcasing that the art of Limenios was not an isolated appearance. Rather, it spanned centuries and different geographical settings. Like the paean of Limenios, which had a vocal counterpart that cannot be perfectly tuned, these compositions also have a vocal counterpart with tuning conflicts.

There is also one additional, relevant source, which may have contained exercises used for training. This source demonstrates that some of the art of highly skilled musicians was shared among a broader group of people. Finally, there are several smaller instrumental fragments, none of which contains tuning conflicts.

While the evidence suggests that pure intonation and perfect tuning were used on lyres, it is also evident beyond doubt that vocal compositions led into tuning conflicts and did not allow for perfect tuning. Thus, a clear musical distinction is found in the compositions encoded in the two notation styles, matching exactly the expectations.

The examples of tuning conflicts in vocal compositions are many. The paean of Athenaios, which is a vocal counterpart to that of Limenios, does not allow for perfect tuning. Similarly, PW

50 has been interpreted as a vocal counterpart to the instrumental pieces PW 51 and 51, and while PW 50 has a tuning conflict, its instrumental counterparts do not. Other examples include the music of Mesomedes, which cannot be tuned perfectly. Such music is not less beautiful. The compositions of Mesomedes have inspired the birth of modern Opera in the late Renaissance. The music that survived in vocal notation is no less well composed, but it is different. Beyond reasonable doubt, this music did not allow for perfect tuning. Theoreticians, such as Ptolemy, further support that music for wind instruments and voice was also perceived to be less precise due to the nature of the instruments.

With these considerations in mind, it is nevertheless wise to continue to investigate and look for additional evidence to enhance the present knowledge of ancient Greek and Roman music.
